# Alteration of cystic airway mesenchyme in congenital pulmonary airway malformation

**DOI:** 10.1038/s41598-019-41777-y

**Published:** 2019-03-28

**Authors:** Yi Jiang, Yongfeng Luo, Yang Tang, Rex Moats, David Warburton, Shengmei Zhou, Jianlin Lou, Gloria S. Pryhuber, Wei Shi, Larry L. Wang

**Affiliations:** 1 Department of Pathology, the Second Xiangya Hospital of Central South University, Changsha, China; 2 Developmental Biology and Regenerative Medicine Program, Children’s Hospital Los Angeles, Los Angeles, CA 90027 USA; 3 Department of Pathology and Laboratory Medicine, Children’s Hospital Los Angeles, Los Angeles, CA 90027 USA; 4 Institute of Occupational Diseases, Zhejiang Academy of Medical Sciences, Hangzhou, China; 5 Department of Pediatrics, University of Rochester School of Medicine and Dentistry, Rochester, NY 14642 USA

## Abstract

Congenital pulmonary airway malformation (CPAM) is the most common congenital lesion detected in the neonatal lung, which may lead to respiratory distress, infection, and pneumothorax. CPAM is thought to result from abnormal branching morphogenesis during fetal lung development, arising from different locations within the developing respiratory tract. However, the pathogenic mechanisms are unknown, and previous studies have focused on abnormalities in airway epithelial cells. We have analyzed 13 excised lung specimens from infants (age < 1 year) with a confirmed diagnosis of type 2 CPAM, which is supposed to be derived from abnormal growth of intrapulmonary distal airways. By examining the mesenchymal components including smooth muscle cells, laminin, and elastin in airway and cystic walls using immunofluorescence staining, we found that the thickness and area of the smooth muscle layer underlining the airway cysts in these CPAM tissue sections were significantly decreased compared with those in bronchiolar walls of normal controls. Extracellular elastin fibers were also visually reduced or absent in airway cystic walls. In particular, a layer of elastin fibers seen in normal lung between airway epithelia and underlying smooth muscle cells was missing in type 2 CPAM samples. Thus, our data demonstrate for the first time that airway cystic lesions in type 2 CPAM occur not only in airway epithelial cells, but also in adjacent mesenchymal tissues, including airway smooth muscle cells and their extracellular protein products. This provides a new direction to study the molecular and cellular mechanisms of CPAM pathogenesis in human.

## Introduction

Congenital pulmonary airway malformation (CPAM), initially referred as a congenital cystic adenomatoid malformation (CCAM)^1^, is a relatively rare developmental anomaly in general, with incidence from 1:11,000 to 1:33,000 live births reported in different countries^2,3^, but it is the most common congenital lesion detected in the neonatal lung. About 25% of neonates with CPAM may present with respiratory distress, infection, and pneumothorax while the rest may be asymptomatic^4^. Those symptomatic patients usually require surgery such as lobectomy to improve respiratory problems and to treat infection and pneumothorax. The perinatal mortality of CPAM ranges from 9% to as high as 49%^5^, and is affected by association with other malformation or hydrops, fetal interventions, and elective termination of pregnancy.

CPAM is thought to result from abnormal lung development. In humans, lung development can be divided into five stages based on anatomic structural characteristics: embryonic (gestation week 3 to 7), pseudoglandular (week 5 to 17), canalicular (week 16 to 26), saccular (week 26 to 36), and alveolar (gestation week 36 to ~postnatal 7 years)^6,7^. Airway branching morphogenesis occurs predominantly in the pseudoglandular stage, during which epithelial airways undergo reiterated division, elongation, and expansion with different growth patterns such as domain, planar, and orthogonal branching^8^. In addition to epithelial cell growth, surrounding mesenchymal cells are also important to this process by providing specific morphogens, extracellular matrix protein-mediated physical supports, and mechanical forces such as airway smooth muscle contraction-induced peristalsis^9^. Therefore, coordinated growth of both epithelial and mesenchymal cell compartments is critical for normal tree-like airway formation, and disruption of this coordinated process results in abnormal lung morphogenesis including congenital airway cystic formation.

CPAM can be divided into 5 types based on its pathological characteristics and the presumed site of malformation^10^: type 0 (trachea/bronchia), type 1 (bronchia/proximal bronchiole), type 2 (distal bronchiole), type 3 (bronchioalveolar region), type 4 (acini). Among them, Type 1 and 2 are the most common forms, which are assumed to originate from intra-pulmonary conducting airways during branching morphogenesis. Histologically, type 2 CPAM has multiple small cysts (<2 cm in diameter) and back-to-back bronchiole-like structures lined by a simple cuboidal to columnar epithelium, while Type 1 CPAM displays larger cysts (>2 cm in diameter) lined with ciliated pseudostratified columnar or simple columnar epithelial cells, with underlying small cartilage islands observed in mesenchyme.

CPAM occurs sporadically. Its formation is not related to particular maternal factors such as race, age, or intrauterine exposures^11,12^. Although a slight male preponderance was reported in several studies with small sample sizes^13–15^, gender predilection is still uncertain^16^. The pathogenic mechanisms by which abnormal lung branching leads to cystic pathology are not completely understood. No defined genetic factors are specifically linked to CPAM pathogenesis^17^. Abnormal increases in cell proliferation and decreases of apoptosis have been reported in some cases of CPAM^18^. In mice, alterations of growth factor pathways in airway epithelial cells, including SHH and YAP-Hippo, cause reduced airway branching with dilated airway tips in fetal lungs^19,20^. A recent study in mice has also suggested that deficiency in developing airway smooth muscle cells (SMCs) around the branching tips prevents terminal bifurcation and cleft formation, resulting in buckling tips^21^. However, there is no animal model that mimics human CPAM pathology with similar cellular and molecular changes, and alteration of airway smooth muscle and other mesenchymal components in human CPAM specimens has not been carefully examined. Thus, further characterization of human CPAM pathology at the cellular and molecular levels, particularly focusing on SMCs and extracellular matrix proteins in airway walls, becomes necessary in order to understand this disease. In this study, among 21 CPAM patient’s samples collected from 2007 to 2016 in our hospital, 13 specimens were selected based on patient age (<1 year old) and the CPAM pathological type (type 2 CPAM only), to avoid confounding effects of developmental stage and type-specific variation in anatomic originating locations.

## Results

### Validation of type 2 CPAM pathology

**Figure 1:**
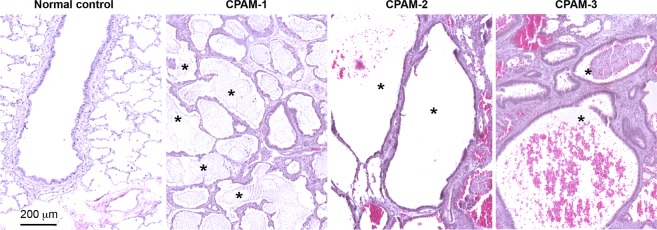
Validation of type 2 CPAM histopathology. Cystic airway structures from three randomly selected CPAM lung specimens (1–3) are shown by H&E staining. Back-to-back bronchiole-like structures are shown by the asterisks. Normal distal bronchiolar structure from a control lung was included for comparison.

The type 2 CPAM histopathologic changes were first validated by microscopy of hematoxylin and eosin (H&E) stained lung sections (Fig. 1). Clusters of small cysts, or so-called back-to-back bronchiole-like structures lined with a simple cuboidal-columnar epithelial layer were evident. No alcian blue-positive mucogenic cells were detected in the cysts (data not included). Underneath the epithelial cells, a thin layer of SMCs and fibrovascular connective tissue were observed in the cystic wall. These histopathologic characteristics are consistent with what have been described for type 2 CPAM^10^, indicating malformation of local airways during fetal lung development.

### Significant reduction of smooth muscle cell layer underlying cystic airway epithelia compared to that in normal bronchioles

**Figure 2:**
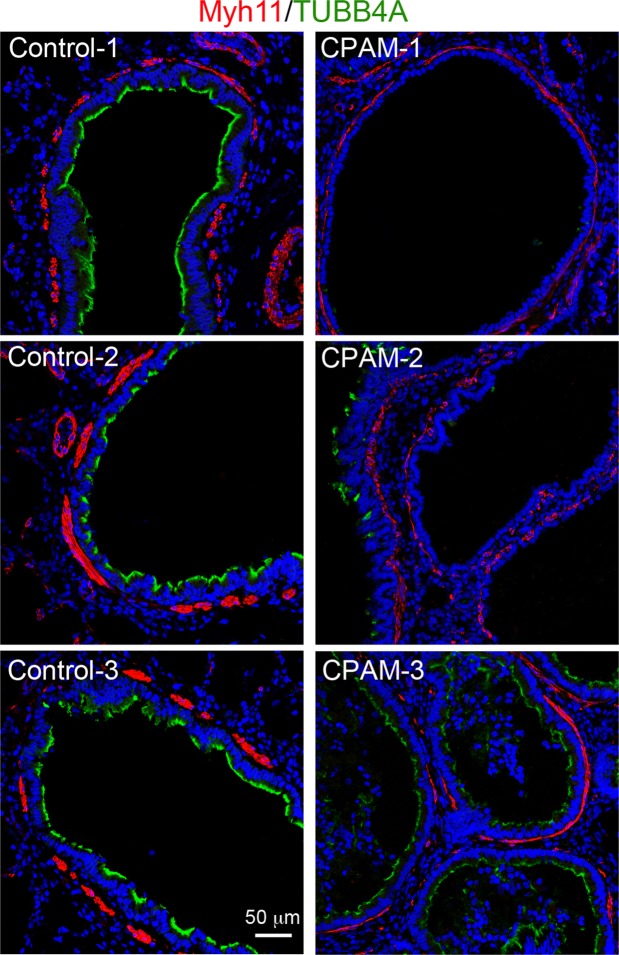
Reduced smooth muscle layers in cystic airway walls of CPAM. Compared to normal bronchiolar walls, the thickness of the smooth muscle cell layer, detected by MYH11 staining (red), is reduced in CPAM samples. Ciliated epithelial cells were co-stained by anti-TUBB4A antibody (green), and cell nuclei were counterstained with DAPI (blue). Images from 3 randomly selected type 2 CPAM specimens and 3 normal lungs are presented.

Recent studies suggest that airway smooth muscle  are likely to be important physiologically in embryonic lung morphogenesis, particularly by physically wrapping the neck/cleft sites of airway tips^21^, and by maintaining positive intraluminal pressure with spontaneous peristaltic contractions^9^. We therefore determined whether the airway smooth muscle layer in type 2 CPAM lungs had qualitatively and/or quantitatively changed. SMCs were specifically stained using an antibody against myosin heavy chain 11 (MYH11, Fig. 2). The smooth muscle layers around normal bronchioles were shown as thick and discontinuous bands. In contrast, the thickness of smooth muscle layers around cystic airways appeared to be significantly reduced compared to the normal bronchioles (Fig. 2). In addition, expression of the ciliated cell marker, TUBB4A (β-tubulin IV), in cystic epithelial cells was also reduced, suggesting abnormal epithelial cell differentiation.

**Figure 3:**
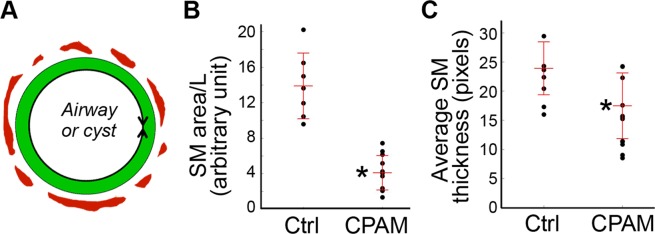
Semi-quantitative measurements and comparisons of airway smooth muscle layers. (**A**) Schematic illustration for average smooth muscle (SM, red) thickness and SM area. The mucous epithelial layer is marked as green. The mucous membrane length (L, shown as a double arrow line) was used for normalization. (**B**) Sum smooth muscle area normalized by mucous membrane length shown as a dot plot, with the bars indicating mean ± se. (**C**) Average smooth muscle layer thickness measurement shown as a dot plot, with mean ± se. (**B**,**C**) Comparisons were performed between cystic airways of type 2 CPAM samples (n = 13) and normal bronchioles in control lungs (Ctrl, n = 7) using non-parametric Mann-Whitney U test. *P < 0.05.

Semi-quantitative measurements were then used to confirm these changes in 2-D tissue sections (Fig. 3). These include the average smooth muscle thickness and sum area of smooth muscle layer normalized by total length of the lined mucous membrane. Comparison was performed between cystic airways and the equivalent bronchioles of normal control lungs. We found that average smooth muscle thickness and normalized smooth muscle area in cystic airways of type 2 CPAMs were all significantly reduced compared to the controls (Fig. 3B,C, P < 0.05). Therefore, reduction of cystic airway smooth muscle layers was evident in our type 2 CPAM specimens.

### Changes of extracellular matrix proteins in cystic airways

**Figure 4:**
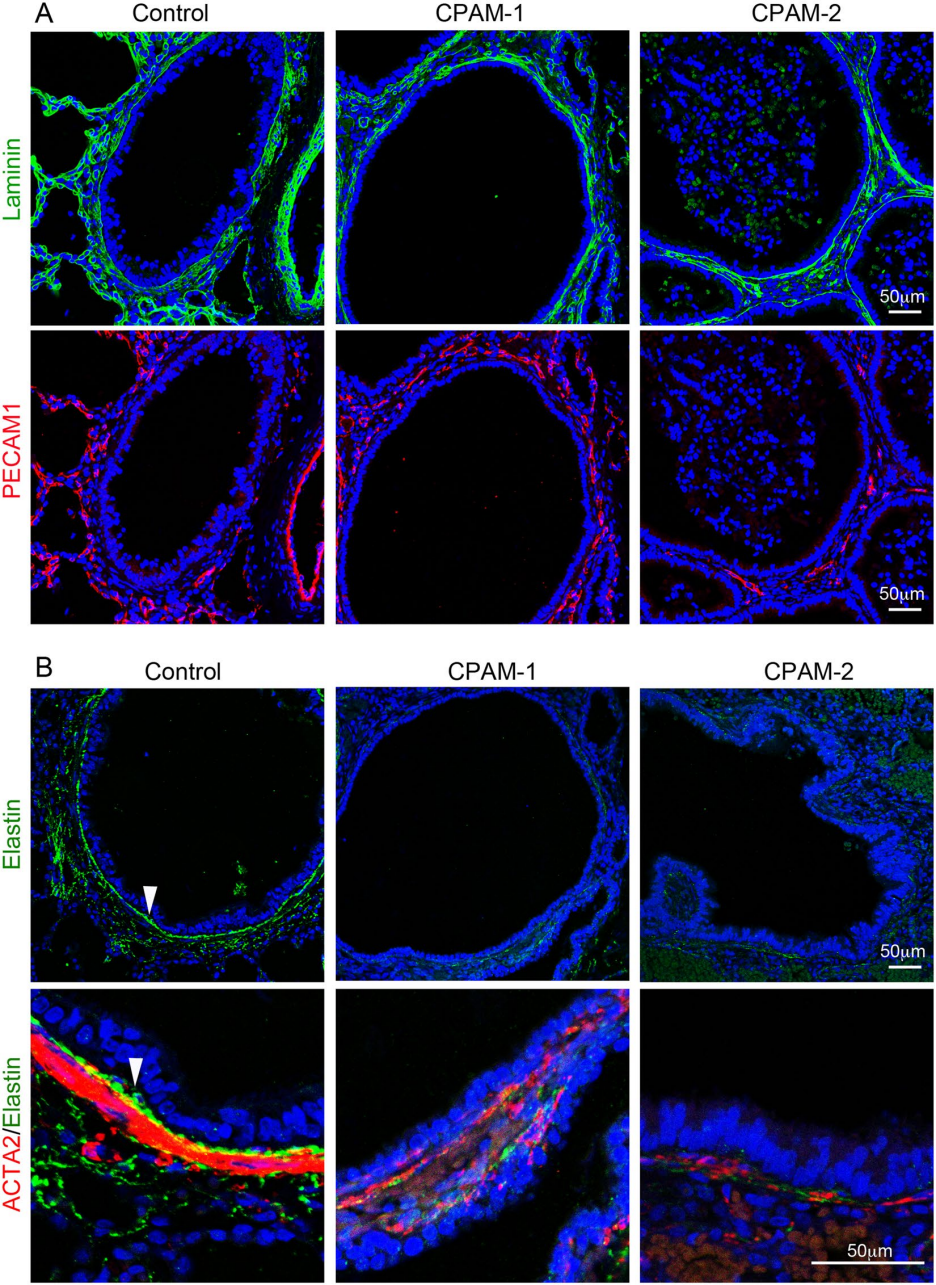
Changes of extracellular matrix proteins in cystic airways of type 2 CPAM. (**A**) Laminin (green) was co-immunostained with vascular endothelial marker PECAM1 (red) and presented in separate panels. (**B**) Elastin (green) was co-immunostained with smooth muscle cell marker ACTA2 (red). Cell nuclei were counterstained with DAPI (blue). The lower panel shows a magnified small area from upper panel. Arrow: An elastin layer between epithelia and SMCs. Control samples were from normal age-matched lungs, and type 2 CPAM samples (CPAM-1 or -2) were from different patients.

To further characterize the pathological changes of CPAM cystic lesions at the molecular level, two major extracellular proteins, laminin and elastin, were examined for their expression and distribution by immunofluorescence staining. Laminin, a major component of basement membrane underneath airway epithelia, vascular endothelia, or SMCs, was detected in cystic airway walls without observable changes (Fig. 4A). Although it has been reported that reduced vascularity was found in CPAM lungs by immunohistochemistry using a CD34 antibody^22^, we did not see consistent changes of vasculature in type 2 CPAM lungs using a PECAM1 antibody (Fig. 4). However, expression of elastin fibers was drastically reduced in the cystic walls compared to the normal airways (Fig. 4B). In particular, a thick elastin band was consistently detected in the submucosal area between epithelia and smooth muscle layers of normal airways, which was absent in cystic type 2 CPAM airways. This suggests that alteration of extracellular matrix proteins may be also involved in cystic formation and/or progression.

## Discussion

CPAM is a congenital pulmonary disorder, resulting from abnormal lung development. The majority of CPAM are caused by abnormal airway branching morphogenesis in fetuses. In our current study, we found that the smooth muscle layer underlining the airway cysts in type 2 CPAM were significantly decreased compared with those in bronchiolar walls of normal controls. Extracellular elastin fibers were also reduced in airway cystic walls, suggesting lung mesenchymal abnormalities.

Mutual interaction between developing lung airway epithelial and surrounding mesenchymal compartments play a key role in controlling airway growth and patterning. Growth factors produced from mesenchymal cells, such as Fgf10, Wnt2/Wnt2b, and Bmp4, are essential for epithelial growth and branching, while growth factors from epithelial cells, such as Shh, Fgf9, and Wnt7b, stimulate the surrounding mesenchymal cells proliferation and differentiation, as well as regulating extracellular matrix production^23^. Alteration of either epithelial or mesenchymal component results in changes in airway branching morphogenesis, presenting as reduced airway number, altered airway size and shape, etc.^23^. The type 2 CPAM phenotype is presumed to originate from abnormal branching of distal bronchioles.

The pathogenic mechanisms of CPAM remain largely unknown, and several potential factors responsible for CPAM pathology are suggested by various studies^24^. Cass *et al*., reported that increased cell proliferation and decreased apoptosis were detected in 12 specimens of CPAM (type 1, 2, and 3) lungs, which also varied between samples obtained at fetal and neonatal stages^18^. Specific epithelial cells in CPAM have been examined, and Club cells, detected by CC10, were reported to increase in fetal CPAM samples by immunostaining or RNA microarray^25,26^. However, increased Club cells were seen in only 2 out of 13 postnatal type 2 CPAM samples in our study (data not included). The change consistently observed in our study of infant type 2 CPAM specimens was in the mesenchyme, where there was a reduction of airway SMCs in the cystic walls.

Lung airway SMCs are differentiated from embryonic lung mesenchymal progenitors^27^. While the physiological role of airway SMCs is not well understood, it is known that contraction and/or proliferation of these cells in the postnatal lung contribute to asthma pathogenesis and progression by limiting air conduction. A recent study in mouse fetal lung development suggests that airway SMCs during branching morphogenesis behave as a girdle around the bifurcating epithelial bud, directing epithelial tip division^21^. Inhibition of airway smooth muscle cell growth disrupts normal epithelial tip bifurcation, resulting in epithelial buckling, possibly by changing physical confining barriers and/or reduction of fetal airway intraluminal pressure. Shh from airway epithelial cells is thought to be a key factor that induces adjacent mesenchymal progenitor cell differentiation into SMCs in mice^28^. Genetic deletion of Shh resulted in failure of branching, forming a small lung with dilated sacs, accompanied by decreased cell proliferation and enhanced cell death^29^. The relationship between defective SHH-airway smooth muscle development and congenital airway cyst formation in human has not been thoroughly investigated. In addition, altered expression of FGF7, FGF9, and PDGFB have been reported in resected CPAM lung samples^30,31^. These growth factors are also important in regulating lung mesenchymal cell growth. Interestingly, adenoviral vector-mediated focal overexpression of FGF10 in the mesenchymal compartment of the fetal rat lung results in macrocystic or microcystic airway malformation, depending on the stage and location of the exogenous FGF10 expression^32^. However, changes of FGF10 and FGFR2 expression were not found in human CPAM samples. Considering the potential difference between rodent and human lungs, direct comparison of these signaling pathways in freshly isolated human lung mesenchymal subtype cells such as SMCs will be critical in understanding the role of these pathways in generating the characteristic CPAM phenotype. We took the first step by examining the relationship between congenital lung cysts and airway SMCs. We report for the first time that congenital airway cystic lesions are characterized by reduction of underlying SMCs in type 2 CPAM lung specimens. Similar changes were also observed in a few cases of type 1 CPAM (data not shown), which needs to be confirmed in more type 1 CPAM samples. Whether reduction of airway SMCs in embryonic lung branching morphogenesis is one of the major mechanisms causing airway cyst formation or a consequence of airway cystic dilation remains to be investigated, particularly in samples harvested at an early stage prior to development of significant cystic lesions. Similarly, investigation of above signaling pathways in lungs at the critical CPAM initiating stage instead of postnatally resected CPAM lung will be critical in determining whether alterations of these pathways contribute to CPAM pathogenesis.

In addition, altered distribution of airway elastin fibers was detected in cystic airways of our type 2 CPAM samples, in particular, a layer of elastin fiber between airway epithelia and smooth muscles was missing. Airway elastin fibers can be produced by adjacent mesenchymal cells including SMCs^33^. In mice, genetic deletion of elastin does not affect airway branching morphogenesis in prenatal lung^34^, which suggests that reduction of elastin fiber in cystic walls may not serve as one of the causative factors for CPAM. However, a defect in elastin distribution around airway SMCs during lung development may also affect smooth muscle cell growth and maintenance as well as mechanical property of airways, which may contribute to cystic progression.

In summary, airway cystic lesions in type 2 CPAM occur not only in airway epithelial cells, but also in adjacent mesenchymal tissues, including airway SMCs and their extracellular matrix proteins such as elastin. Therefore, further detailed molecular and cellular studies using both human samples combined with *in vivo* dynamic disease models are needed in order to understand CPAM pathogenic mechanisms.

## Materials and Methods

### CPAM specimens

Surgically excised lung tissues were fixed in 4% buffered formalin solution and embedded in paraffin blocks as a standardized operating procedure in the Department of Pathology at Children’s Hospital Los Angeles. Lung tissue sections from 13 type 2 CPAM patients, who were diagnosed from 2007 to 2016 by the Department of Pathology, Children’s Hospital Los Angeles, were used in this study. All patients were ≤1 year old (n = 13, mean ± se: 5.23 ± 1.29 months), including both males (n = 6) and females (n = 7). Normal lung control samples (n = 7, mean ± se: 2.29 ± 0.96 months, P > 0.05 compared to patients) were obtained from autopsy, provided by the Biorepository For Investigation of Neonatal Diseases of the Lung at University of Rochester. Informed consent was obtained from the parents or legal guardians, and experimental protocols were approved by the Institutional Review Board of Children’s Hospital Los Angeles. All methods were carried out in accordance with relevant guidelines and regulations.

### Histology and immunofluorescence analysis

4 μm-thick serial paraffin sections were used for both histopathology and immunofluorescence staining studies. H&E staining was used for routine lung histologic structural examination. Cell types were visualized by immunofluorescence staining for cell-specific molecular markers, including ACTA2 (A2547, mouse monoclonal antibody (clone 1A4) with 1:1000 dilution, Sigma-Aldrich) for SMCs and myofibroblasts^35^, MYH11 (Ls-B9445, rabbit monoclonal antibody (EPR5335) with 1:400 dilution, LSBio) for SMCs^36^, PECAM1 (sc-1506, goat polyclonal antibody with 1:1000 dilution, Santa Cruz) for endothelial cells^37^, TUBB4A (MU178-UC, mouse monoclonal antibody (ONS1A6) with 1:1000 dilution, BioGenex) for ciliated airway epithelial cells^35^. In addition, key extracellular matrix proteins including elastin and laminin were also examined. Rabbit anti-elastin polyclonal antibody (1:1000 dilution) was provided by Dr. Robert Mecham at Washington University St. Louis^37,38^, and rabbit polyclonal anti-pan-laminin antibody was purchased from Thermo Scientific (#RB-082, 1:1000 dilution)^37^. Cell nuclei were counterstained with DAPI. Antigen retrieval was performed for the rehydrated tissue sections by boiling the slides in Tris-EDTA buffer (10 mM Tris, 1 mM EDTA, 0.05% Tween 20, pH 9.0) for 30 min prior to immunofluorescence staining. After blocking with 5% bovine serum albumin-1% donkey serum, the tissue sections were incubated with primary antibodies overnight at 4 °C. Following extensive washing, the tissue sections were then incubated with fluorescence-labeled secondary antibodies (donkey anti-rabbit, anti-mouse, and anti-goat antibodies) for 1 hour at room temperature. Images were captured using Zeiss LSM710 confocal microscope at the Imaging Core Facility of the Saban Research Institute of Children’s Hospital Los Angeles as described in our previous publication^27^.

### Semi-quantitative airway smooth muscle layer analysis

In 2-D human lung tissue sections, distal bronchioles with a diameter of 200–500 μm or cysts were selected for analyzing the SMCs underlying the epithelial layer. Five randomly selected fields per tissue section were imaged under 100X magnification, and three different sections per sample were used for immunostaining. Images were analyzed using counter plugin of Image J software. Smooth muscle length of the cyst or airway cross-section was measured as the sum of all smooth muscle lengths along the airways or airway cysts. Average airway smooth muscle thickness was the mean of smooth muscle layer thickness of each airway or airway cyst. Airway smooth muscle area was then calculated. The lined airway mucous membranes perimeter length was used to normalize the values for airway smooth muscle area (see Results for detail).

### Statistical analysis

All quantitative data were presented as mean ± se. Non-parametric Mann-Whitney U test (two-tailed) was used for statistical comparison between control group and type 2 CPAM group, with P < 0.05 considered significant.
